# USING COMMUNITY-BASED PARTICIPATORY RESEARCH TO PROMOTE MENTAL HEALTH LITERACY IN OLDER CHINESE

**DOI:** 10.1093/geroni/igac059.2677

**Published:** 2022-12-20

**Authors:** Jessie Ho-Yin Yau, Edwin Lok, Yan Wong, Hotinpo Sky Kanagawa, Nicole H L Wong, Tianyin Liu, Gloria H Y Wong, Terry Lum

**Affiliations:** The University of Hong Kong, Hong Kong, Hong Kong; The University of Hong Kong, Hong Kong, Hong Kong; The University of Hong Kong, Hong Kong, Hong Kong; The University of Hong Kong, Hong Kong, Hong Kong; The University of Hong Kong, Hong Kong, Hong Kong; The University of Hong Kong, Hong Kong, Hong Kong; The University of Hong Kong, Hong Kong, Hong Kong; The University of Hong Kong, Hong Kong, Hong Kong

## Abstract

Whilst traditional mental health literacy programmes utilized a top-down approach, no bottom-up community-based participatory research (CBPR) model had been used to promote mental health literacy among older adults. Moreover, the existing CBPR model was developed in the West and might not be applicable in Chinese communities given different socio-cultural contexts. This research aimed to fill the gap by implementing a CBPR project to promote mental health literacy among older adults and developing a CBPR model in Chinese context. A year-long CBPR project was conducted in five Hong Kong districts from May 2021 to August 2022. A district advisory group was formed in each district, which comprised 50 community older adults, 2 NGO social workers, and 2 researchers. Each district had their own promotional activities that were initiated and designed by older adults, including street booths, art workshops, videos and photos to promote mental health and introduce relevant information and resources. Researchers recorded field observations in each district activity and conducted focus group discussions with stakeholders. Collected data suggested that specific elements are important for a CBPR model in Chinese context, including participant empowerment, technical support, stakeholder expectation management and potential community contribution. Following the implementation of district promotional activities, a territory-wide advisory group was formed to promote mental health literacy on a territory level in the coming year. This is the first large-scale CBPR project that promoted mental health literacy among older adults in Chinese context. Implications for future research and practice will be discussed.

